# Internal Limiting Membrane Flap in the Management of Retinal Detachment due to Paracentral Retinal Breaks

**DOI:** 10.1155/2019/4303056

**Published:** 2019-01-21

**Authors:** Yen-Chih Chen, Chung-May Yang, San-Ni Chen

**Affiliations:** ^1^Department of Ophthalmology, Changhua Christian Hospital, Changhua, Taiwan; ^2^School of Medicine, National Taiwan University, Taipei, Taiwan; ^3^School of Medicine, Chung-Shan Medical University, Taichung, Taiwan; ^4^School of Medicine, Kaohsiung Medical University, Kaohsiung, Taiwan; ^5^Department of Optometry, Da-Yeh University, Changhua, Taiwan

## Abstract

**Purpose:**

To describe the technique and outcomes of using either inverted or free internal limiting membrane flap in the management of retinal detachment due to paracentral retinal breaks.

**Methods:**

This retrospective observational case series includes nine patients who received surgery for retinal detachment due to paracentral retinal breaks developed either from primary rhegmatogenous origin, or secondary iatrogenic retinal breaks after prior membrane peeling, or during surgery for tractional retinal detachment. Either inverted or free internal limiting membrane flaps were inserted in the identified breaks, followed by air fluid exchange and gas tamponade. Visual acuity and structural changes were evaluated.

**Results:**

Nine eyes were included. One had primary rhegmatogenous retinal detachment, one had highly myopic eye with peripapillary atrophic hole, three had secondary retinal detachment after membrane peeling for foveoschisis or macular pucker, one had recurrent retinal detachment due to proliferative vitreoretinopathy, one had combination of tractional and rhegmatogenous retinal detachment, and two had iatrogenic breaks during surgery. The retinal breaks of all eyes were sealed with retina attached postoperatively. Visual acuity in logarithm of minimal angle of resolution improved from 1.18 ± 0.55 preoperatively to 0.74 ± 0.47 postoperatively (*p*=0.04).

**Conclusion:**

Internal limiting membrane flap technique can be a surgical approach selectively for retinal detachment due to paracentral retinal breaks with difficulty for laser application. The retina can be attached successfully and achieve good visual outcome without major complication. This trial is registered with NCT03707015.

## 1. Introduction

Retinal detachment (RD) due to posterior paracentral retinal breaks is a rare condition. In contrary to macular hole associated retinal detachment (MHRD), which is mostly often observed primarily in highly myopic eyes, posterior paracentral retinal breaks are often secondary. These breaks are either paravascular or juxtapapillary breaks related to pathologic myopia [1, 2] or are found in complicated cases of proliferative diabetic retinopathy (PDR), branch retinal vein occlusion [3], or combined tractional and rhegmatogenous retinal detachment (CTRRD). Besides, iatrogenic paracentral retinal breaks have also been reported after membrane peeling in macular surgery [4, 5] or tractional retinal detachment (TRD).

To treat RD due to posterior breaks, pars plana vitrectomy with air fluid exchange, accompanied by laser retinopexy around the breaks and either gas or silicone oil tamponade nowadays, are mostly used [6, 7]. However, laser retinopexy around those paracentral breaks is difficult to perform in complicated cases owing to the retinal pathology. Besides, laser photocoagulation within posterior poles may lead to a certain degree of permanent visual field defects [8, 9].

The inverted internal limiting membrane (ILM) flap technique has been increasingly used for refractory macular hole (MH) [10–14] and MHRD [15] with successful surgical outcomes and high closure rates. Free ILM flap technique has also been shown to be effective in closing recalcitrant macular holes [16]. We therefore used ILM flap technique, either by inverted ILM flap or free ILM flap to close the paracentral breaks and treat the associated retinal detachment. In this study, we reported the surgical outcomes of eyes with RD due to posterior paracentral retinal breaks by using ILM flap technique without laser retinopexy.

## 2. Materials and Methods

This was a retrospective, consecutive case-series study conducted at Changhua Christian Hospital. The study was approved by the Ethics Committee and Research Board of Changhua Christian Hospital, and all procedures adhered to the Declaration of Helsinki. Informed consent was obtained from each patient.

We included patients with RD and posterior paracentral retinal breaks located within the equator from January 2017 to January 2018. Eyes with macular hole were excluded. All of the cases underwent standard pars plana vitrectomy using the ILM flap technique, accompanied by extended gas tamponade without laser retinopexy. All operations were performed by a single experienced vitreoretinal surgeon (SN Chen).

All of the patients underwent thorough ophthalmological examinations including slit lamp and indirect ophthalmoscopy examinations, color fundus photography, and spectral-domain optical coherence tomography (OCT) (Heidelberg Spectralis, Germany) preoperatively and postoperatively. Data including age, gender, and Snellen best-corrected visual acuity (BCVA) were recorded. Infrared-autofluorescence imaging (HRA, Retinal Angiograph; Heidelberg Engineering, Heidelberg, Germany) and spectral-domain OCT were used postoperatively to localize the retinal breaks. All of the patients were followed up at outpatient clinics for at least 3 months after the surgery.

### 2.1. Surgical Technique

Pars plana vitrectomy was performed in all patients with a 23-gauge vitrectomy probe using a constellation system (Alcon Laboratories, Inc., Fort Worth, TX). After core vitrectomy and trimming of peripheral vitreous, cortical vitreous stripping with or without triamcinolone acetonide assistance was performed within the arcade and around the breaks in all eyes. The ILM was then stained with indocyanine green (ICG) solution (25 mg ICG in 15 ml 5% glucose-water solution, final concentration = 1.7 mg/ml) followed by subretinal fluid drainage through the posterior breaks with soft tip until the retina was almost attached. The ILM flap was then prepared by first peeling ILM in a circular fashion to create an ILM island. The size of the ILM island was dependent on the size of retinal break, and the diameter of ILM island was at least twice than that of the break. ILM was peeled with a hinge attached to the break. Then, the inverted ILM flap was inserted into the breaks. Basically, total ILM peeling around the breaks was performed if possible but for those breaks with difficulty in peeling the surrounding ILM or peeling as an inverted flap, we harvest free ILM flap in a whole piece from other site within posterior pole. Then the free ILM flap was transplanted into the breaks.

An incomplete air-fluid exchange was then performed. Pan-retinal photocoagulation was performed in eyes with PDR. Sulfur hexafluoride (SF_6_) with a concentration from 20% to 40% was infused in seven eyes, and perfluoropropane (C_3_F_8_) with a concentration from 7% to 20% was infused in two eyes. The concentration of gas depended on the residual amount of subretinal fluid at the end of surgery. The patients were instructed to maintain a prone position after the operation for 1 week (see supplemental digital content for the video (available here)).

### 2.2. Statistical Analysis

Snellen BCVA was converted to the logarithm of minimal angle of resolution (LogMAR) for statistical analysis. Preoperative and postoperative BCVA values were compared using the Wilcoxon signed-rank test for nonparametric analysis. All analyses were performed using MedCalc software version 18 (MedCalc Software, Mariakerke, Belgium).

## 3. Results

There were nine consecutive cases (five males and four females) with an average age of 56.89 ± 9.61 years (range, 45 to 74 years).

**Figure 1:**
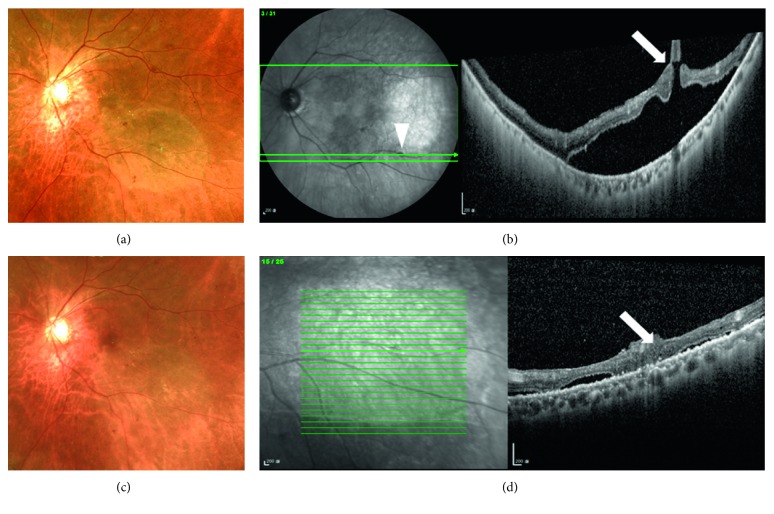
A 67-year-old male patient (case 1) with shadow vision in the left eye for 1 week with a corrected visual acuity of 20/60. Primary rhegmatogenous retinal detachment (RRD) of the posterior pole of the left eye was noted at our clinic (a). The break was located within vascular arcade, near the inferior temporal vessel and well identified by preoperative optical coherence tomography (OCT, b). He underwent vitrectomy with inverted internal limiting membrane (ILM) flap technique, accompanied by 30% sulfur hexafluoride infusion. Two weeks postoperatively after the absorption of air, the retina was well attached (c). Magnified OCT showed that the break had sealed, the presence of the ILM flap, and absorption of the subretinal fluid (d). His visual acuity improved to 20/40 (see supplemental digital content of the video (available here)).

Two cases had primary rhegmatogenous retinal detachment (RRD) (cases 1 and 2, Figure 1) including one with highly myopic eye with a peripapillary atrophic hole.

**Figure 2:**
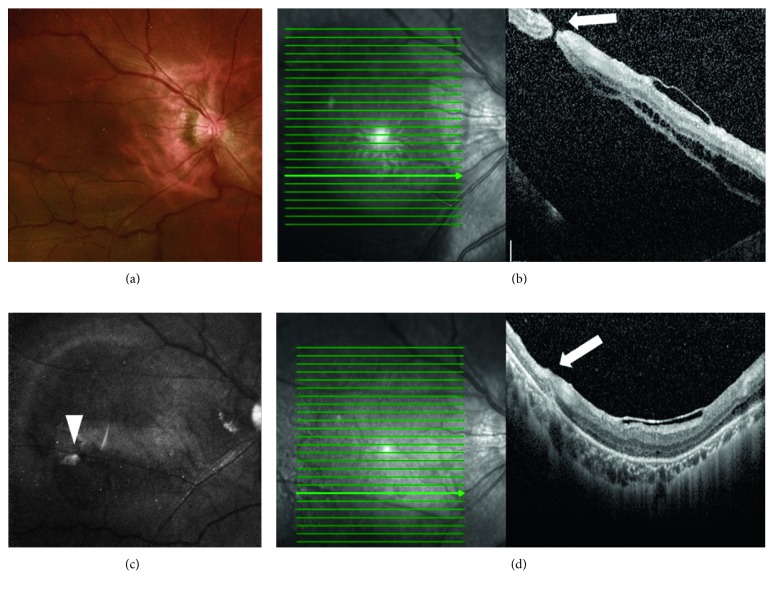
A 46-year-old highly myopic male patient (case 8) had a previous history of vitrectomy, internal limiting membrane (ILM) peeling, and semicircular ILM flap over the fovea for foveoschisis in his right eye. He developed retinal detachment over his right eye 2 months later (a). Preoperative optical coherence tomography (OCT) showed a small break located temporally within the posterior pole (b). After vitrectomy, an inverted ILM flap was made and inserted into the small break with 24% sulfur hexafluoride gas tamponade. The retina was found to be reattached 2 weeks later, and infrared autofluorescence imaging showed a hyperfluorescent spot of the ILM plug corresponding to the previous retinal break (c). Postoperative OCT confirmed ILM tissue at the site of the previous break (d).

Three had secondary RRD after membrane peeling for either macular pucker or foveoschisis (cases 3, 8, and 9; Figure 2).

**Figure 3:**
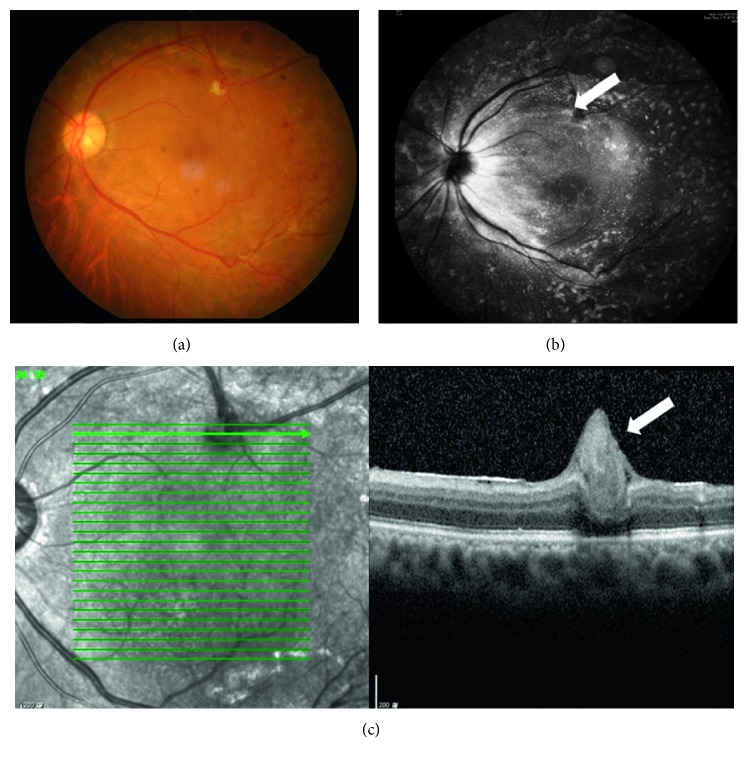
A 57-year-old male patient (case 6) with proliferative diabetic retinopathy underwent vitrectomy for vitreous hemorrhage and tractional retinal detachment. During surgery, an iatrogenic break was made during the delamination process. After internal limiting membrane (ILM) peeling, a free ILM flap was inserted into the break, followed by panretinal retinal photocoagulation without using the laser around the break, and 24% sulfur hexafluoride gas tamponade. Two weeks later, fundus photography showed that the retina was attached (a). Follow-up optical coherence tomography and infrared autofluorescence photography showed ILM tissue over the break area (b, c).

Two had intraoperative iatrogenic breaks during the surgery for TRD (cases 4 and 6, Figure 3).

One had CTRRD (case 5), and one had recurrent RD with PVR (case 7).  Table 1 shows the demographic data of the patients.

The inverted ILM flap could only be performed in two eyes (cases 1 and 8). Free ILM flap was performed in the other 7 eyes, because the inverted ILM flap could not be achieved in the following conditions: first, for eyes with previous ILM peeling, there is no ILM around the breaks (cases 3 and 9). Second, for a highly myopic eye with peripapillary break and extremely adherent vitreous around the breaks, the vitreous could be peeled (case 2), and third, eyes of TRD with iatrogenic breaks (cases 4 and 6) or for eyes of CTRRD 9 (cases 5 and 7), in which the retina surface is rigid and uneven around the breaks, an inverted ILM flap is impossible to be obtained. The free ILM flaps were harvested within the macular area in 5 eyes (cases 2–7). In the other 2 eyes (cases 3 and 9), because of the previous extensive ILM peeling within the arcade, the free ILM flaps were harvested at an area outside the arcade, where residual ILM was feasible. The retina was successfully reattached in all eyes, and fundus examinations and OCT confirmed that the retinal breaks had been sealed. No major complication was observed postoperatively. The average visual acuity in LogMAR statistically significantly improved from 1.18 ± 0.55 preoperatively to 0.74 ± 0.47 postoperatively (*p*=0.04). The mean follow-up duration was 5.33 ± 2.00 months.

## 4. Discussion

Several methods had been proposed to manage RD due to posterior retinal breaks, including pars plana vitrectomy with silicone oil or gas tamponade, macular buckles [17], and transscleral diathermy [18]. Internal approaches using vitrectomy, air-fluid exchange, laser photocoagulation around the breaks, and gas or silicone oil tamponade remain the mostly widely used procedures [6, 7, 19]. However, progressive enlargement of laser scars with subsequent involvement of the central visual field is the major concern. In addition, laser retinopexy around the fragile, already thin posterior retina in highly myopic eyes and in eyes with PDR, may lead to atrophic holes at the laser margin later. Furthermore, in highly myopic eyes with diffuse atrophic patches and in eyes with the tractional component of CTRRD or TRD, the retina is rigid and difficult to flatten intraoperatively. Laser retinopexy is difficult to apply, requiring heavy energy levels and the use of silicone oil for tamponade. In the current pilot study, all nine patients received the ILM flaps insertion without laser retinopexy and had breaks sealed as well as reattached retina postoperatively for at least 3 months. There are several other advantages of using ILM flap to repair the paracentral retinal holes over laser photocoagulation. First, by using laser, the retinal breaks are sealed, but not actually closed. ILM flap, on the other hand, acting as a scaffold tissue, would bring the glial tissue to cover the retinal breaks and recover the retinal anatomic structure better. Previous studies have shown that even with recurrent retinal detachment, the previous macular hole repaired by inserted ILM tissue remained closed [20–23]. Second, retinal holes with surrounding laser have more chance to have the intravitreal migration of retinal pigment epithelial cells from exposed retinal pigment epithelium and subsequent proliferative changes [24].

In this study, we found that it is not always possible to peel ILM as an inverted flap around the retinal breaks. Thus, most cases in our series have free ILM flap transplantation. Recently, we demonstrated that in cases with MHRD, multiple pieces of free ILM flap insertion into macular hole could efficiently close the macular hole and reattach the retina without the assistance of perfluorocarbon liquid [25]. Rizzo et al. [26] also used free ILM fragment transplantation for paravascular or juxtapapillary breaks associated retinal detachment with very good surgical outcome.

In this study, we used larger ILM flap and then inserted the flap to plug into the breaks without the assistance of perfluorocarbon. This proved to be effective, as infrared autofluorescence localization and OCT showed that the ILM flaps stayed in place during subsequent follow-up in all of our cases. Instead of silicone oil tamponade, either sulfur hexafluoride or perfluoropropane was used in our cases which was reabsorbed spontaneously within 2 weeks. It indicated that the glial tissue would grow over the flap to seal those paracentral breaks within weeks and only gas tamponade is sufficient.

We also noted in some cases that ILM peeling or even cortical vitreous removal around the retinal breaks is not applicable. It is quite different from the previous reported methods in treating MHRD, in which ILM peeling around macular hole was always performed, regardless of using the inverted ILM technique or the free ILM flap technique. In our cases of using free ILM flaps due to difficulty in peeling cortical vitreous and ILM around the breaks, we found the breaks could still be closed. It may indicate that healing of retinal paracentral breaks assisted by free ILM flaps may be strong enough to counteract the tractional force of the remaining ILM and cortical vitreous.

In this study, incomplete air-fluid exchange was used to prevent the residual subretinal fluid gaining access into intravitreal space, pushing and displacing the inverted or free ILM flap away from the retinal breaks. Therefore, gas with expansile concentration was used dependenting on the amount of residual subretinal fluid. The most common postoperative complication in this study was ocular hypertension. Four eyes (cases 1–3 and 7) had transient IOP elevations several days after the surgery due to gas expansion. The elevated IOP returned to normal within 2 weeks after onset.

Recently, Park et al. [27] compared the surgical outcome using insertion ILM technique versus inverted ILM flap in treating macular hole, and they concluded the inverted ILM flap technique has better anatomical recovery and postoperative visual acuity. In our study, we used ILM flap for paracentral breaks rather than macular hole, and the main purpose is to seal the break. For paracentral breaks, the disadvantage of using ILM insertion technique is not the main concern and we showed both free ILM flap insertion and inverted ILM technique effective in sealing retinal breaks and making retina reattached.

The limitation of this study includes the retrospective nature, small number of cases, and short period of follow up. In addition, there are several limitations to this technique. First, this technique could only be used for small retinal breaks. It is more technically challenging to harvest a large sheet of ILM as a whole piece on detached retina. Second, it is sometimes difficult to harvest enough ILM tissue in eyes with previous ILM peeling. Third, although there are good surgical outcome in our study, we did not compare the different ILM methods in the break closure rate owing to the small number of case using the inverted ILM flap. A larger number of cases and a longer follow-up period are necessary to elucidate the efficacy and potential complications in long-term follow-up.

In conclusion, using ILM flap offers some advantages over traditional laser retinopexy, by recovering anatomical structure and preventing complications from laser photocoagulation. Overall, the surgical outcome by using the ILM flap in management retinal detachment due to posterior paracentral retinal breaks is satisfying, with significant visual acuity improvement.

## Figures and Tables

**Table 1:** Demographic data of patients.

Case/age/sex/eye	Retinal detachment type	Tamponade	Best-corrected visual acuity in LogMAR		Duration of follow-up (months)
			Initial	Final	
1/67/M/OS	Primary RRD	30% SF_6_	0.5	0.3	6
2/74/M/OD	Primary RRD (high myopia)	40% SF_6_	2	0.7	3
3/51/F/OS	Secondary RRD (post ILM peeling)	40% SF_6_	0.7	0	5
4/45/F/OD	PDR + TRD + iatrogenic intraoperative break	7% C_3_F_8_	0.8	1.3	3
5/63/M/OS	CTRRD (PDR)	24% SF_6_ + blood	1	0.7	6
6/57/M/OS	PDR + TRD + iatrogenic intraoperative break	22% SF_6_	1	0.4	3
7/54/F/OS	Recurrent RRD with PVR	20% C_3_F_8_	2	1.3	6
8/46/M/OD	Secondary RRD (post ILM peeling)	24% SF_6_ + blood	1.6	1.3	8
9/55/F/OD	Secondary RRD (post ILM peeling)	24% SF_6_	1	0.7	8

C_3_F_8_: perfluoropropane; CTRRD: combined tractional and rhegmatogenous retinal detachment; F: female; ILM: internal limiting membrane; M: male; PDR: proliferative diabetic retinopathy; PVR: proliferative vitreoretinopathy; RRD: rhegmatogenous retinal detachment; SF_6_: sulfur hexafluoride; TRD: tractional retinal detachment.

## Data Availability

No data were used to support this study.

## Supplementary Materials

Supplementary Materials Supplementary material 1: this video demonstrated the surgical procedure of using internal limiting membrane (ILM) flap for rhegmatogenous retinal detachment of our patient (case 1). After vitrectomy, the break and associated retinal detachment was well identified. Indocyanie green (ICG) staining and ILM peeling in an inverted flap fashion were performed. The ILM inverted flap was then inserted into the break, and the correct position of the flap was confirmed after air-fluid exchange. No laser retinopexy for the break was performed. Then, 30% sulfur hexafluoride (SF_6_) was infused. The patients were instructed to maintain a prone position after the operation for 1 week.

## References

[1] Chen L., Wang K., Esmaili D., Xu G. (2010). Rhegmatogenous retinal detachment due to paravascular linear retinal breaks over patchy chorioretinal atrophy in pathologic myopia. *Archives of Ophthalmology*.

[2] Freund K. B., Ciardella A. P., Yannuzzi L. A. (2003). Peripapillary detachment in pathologic myopia. *Archives of Ophthalmology*.

[3] Singh M., Dhir L., Kon C., Rassam S. (2005). Tractional retinal break and rhegmatogenous retinal detachment consequent to branch retinal vein occlusion. *Eye*.

[4] Steven P., Laqua H., Wong D., Hoerauf H. (2006). Secondary paracentral retinal holes following internal limiting membrane removal. *British Journal of Ophthalmology*.

[5] Sandali O., El Sanharawi M., Basli E. (2012). Paracentral retinal holes occurring after macular surgery: incidence, clinical features, and evolution. *Graefe’s Archive for Clinical and Experimental Ophthalmology*.

[6] Yang C.-M., Su P.-Y., Yeh P.-T., Chen M.-S. (2008). Combined rhegmatogenous and traction retinal detachment in proliferative diabetic retinopathy: clinical manifestations and surgical outcome. *Canadian Journal of Ophthalmology*.

[7] Huang C.-H., Hsieh Y.-T., Yang C.-M. (2017). Vitrectomy for complications of proliferative diabetic retinopathy in young adults: clinical features and surgical outcomes. *Graefe’s Archive for Clinical and Experimental Ophthalmology*.

[8] Subash M., Comyn O., Samy A. (2016). The effect of multispot laser panretinal photocoagulation on retinal sensitivity and driving eligibility in patients with diabetic retinopathy. *JAMA Ophthalmology*.

[9] Fong D. S., Girach A., Boney A. (2007). Visual side effects of successful scatter laser photocoagulation surgery for proliferative diabetic retinopathy. *Retina*.

[10] Michalewska Z., Michalewski J., Adelman R. A., Nawrocki J. (2010). Inverted internal limiting membrane flap technique for large macular holes. *Ophthalmology*.

[11] Kuriyama S., Hayashi H., Jingami Y., Kuramoto N., Akita J., Matsumoto M. (2013). Efficacy of inverted internal limiting membrane flap technique for the treatment of macular hole in high myopia. *American Journal of Ophthalmology*.

[12] Michalewska Z., Michalewski J., Dulczewska-Cichecka K., Nawrocki J. (2014). Inverted internal limiting membrane flap technique for surgical repair of myopic macular holes. *Retina*.

[13] Shin M. K., Park K. H., Park S. W., Byon I. S., Lee J. E. (2014). Perfluoro-n-Octane-assisted single-layered inverted internal limiting membrane flap technique for macular hole surgery. *Retina*.

[14] Chen S.-N. (2017). Large semicircular inverted internal limiting membrane flap in the treatment of macular hole in high myopia. *Graefe’s Archive for Clinical and Experimental Ophthalmology*.

[15] Chen S.-N., Yang C.-M. (2016). Inverted internal limiting membrane insertion for macular hole-associated retinal detachment in high myopia. *American Journal of Ophthalmology*.

[16] Morizane Y., Shiraga F., Kimura S. (2014). Autologous transplantation of the internal limiting membrane for refractory macular holes. *American Journal of Ophthalmology*.

[17] Ma J., Li H., Ding X., Tanumiharjo S., Lu L. (2017). Effectiveness of combined macular buckle under direct vision and vitrectomy with ILM peeling in refractory macular hole retinal detachment with extreme high axial myopia: a 24-month comparative study. *British Journal of Ophthalmology*.

[18] Bovey E. H., Gonvers M. (1999). Transscleral diathermy. *Retina*.

[19] Hsu Y. J., Hsieh Y. T., Yeh P. T., Huang J. Y., Yang C. M. (2014). Combined tractional and rhegmatogenous retinal detachment in proliferative diabetic retinopathy in the anti-VEGF era. *Journal of Ophthalmology*.

[20] Hainsworth D. P., Johnson M. W., Jaffe G. J. (1997). Sustained closure of surgically repaired macular holes after retinal detachment with submacular fluid. *American Journal of Ophthalmology*.

[21] Heier J. S., Topping T. M., Frederick A. R., Morley M. G., Millay R., Pesavento R. D. (1999). Visual and surgical outcomes of retinal detachment following macular hole repair. *Retina*.

[22] Tabandeh H., Chaudhry N. A., Smiddy W. E. (1999). Retinal detachment associated with macular hole surgery. *Retina*.

[23] Herring J. H., Chen C. J., Chen L. L. (2000). Confirmation of persistent closure of surgically repaired macular hole in subsequent retinal detachment by optical coherence tomography. *Ophthalmic Surgery, Lasers*.

[24] Tosi G. M., Marigliani D., Romeo N., Toti P. (2014). Disease pathways in proliferative vitreoretinopathy: an ongoing challenge. *Journal of Cellular Physiology*.

[25] Chen S.-N., Hsieh Y.-T., Yang C.-M. (2018). Multiple free internal limiting membrane flap insertion in the treatment of macular hole-associated retinal detachment in high myopia. *Ophthalmologica*.

[26] Rizzo S., Tartaro R., Barca F., Bacherini D., Franco F., Caporossi T. (2018). Autologous internal limiting membrane fragment transplantation for rhegmatogenous retinal detachment due to paravascular or juxtapapillary retinal breaks over patchy chorioretinal atrophy in pathologic myopia. *Retina*.

[27] Park J. H., Lee S. M., Park S. W., Lee J. E., Byon I. S. (2018). Comparative analysis of large macular hole surgeries using an internal limiting membrane: insertion technique versus inverted flap technique. *British Journal of Ophthalmology*.

